# Role of the amygdala‐medial orbitofrontal relationship in odor recognition in the elderly

**DOI:** 10.1002/brb3.2956

**Published:** 2023-03-10

**Authors:** Kei Sakikawa, Yuri Masaoka, Motoyasu Honma, Akira Yoshikawa, Masaki Yoshida, Sawa Kamimura, Masahiro Ida, Hitome Kobayashi, Masahiko Izumizaki

**Affiliations:** ^1^ Department of Physiology Showa University School of Medicine Tokyo Japan; ^2^ Department of Otorhinolaryngology Head and Neck Surgery Showa University School of Medicine Tokyo Japan; ^3^ Division of Health Science Education Showa University School of Nursing and Rehabilitation Sciences Yokohama Japan; ^4^ Department of Ophthalmology Jikei Medical University Tokyo Japan; ^5^ Department of Radiology National Hospital Organization Mito Medical Center Ibaraki Japan

**Keywords:** amygdala, elderly, olfaction recognition, orbitofrontal

## Abstract

**Introduction:**

In patients with mild cognitive impairment, pathological changes begin in the amygdala (AMG) and hippocampus (HI), especially in the parahippocampal gyrus and entorhinal cortex (ENT). These areas play an important role in olfactory detection and recognition. It is important to understand how subtle signs of olfactory disability relate to the functions of the above‐mentioned regions, as well as the orbitofrontal cortex (OFC). In this study, we evaluated brain activation using functional magnetic resonance imaging (fMRI), performed during the presentation of olfactory stimuli (classified as “normal odors” not inducing memory retrieval), and investigated the relationships of the blood oxygen level‐dependent (BOLD) signal with olfactory detection and recognition abilities in healthy elderly subjects.

**Methods:**

Twenty‐four healthy elderly subjects underwent fMRI during olfaction, and raw mean BOLD signals were extracted from regions of interest, including bilateral regions (AMG, HI, parahippocampus, and ENT) and orbitofrontal subregions (frontal inferior OFC, frontal medial OFC, frontal middle OFC, and frontal superior OFC). Multiple regression and path analyses were conducted to understand the roles of these areas in olfactory detection and recognition.

**Results:**

Activation of the left AMG had the greatest impact on olfactory detection and recognition, while the ENT, parahippocampus, and HI acted as a support system for AMG activation. Less activation of the right frontal medial OFC was associated with good olfactory recognition. These findings improve our understanding of the roles of limbic and prefrontal regions in olfactory awareness and identification in elderly individuals.

**Conclusion:**

Functional decline of the ENT and parahippocampus crucially impacts olfactory recognition. However, AMG function may compensate for deficits through connections with frontal regions.

## Graphical abstract

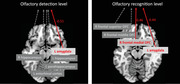

## 1 INTRODUCTION

The amygdala (AMG) plays an important role in olfactory perception (Allison, 1954; Crosby & Humphrey, 1941). Olfactory information is delivered directly to the AMG, which is part of the primary olfactory cortex, from the olfactory bulb (Carmichael et al., 1994). The AMG is divided into three subregions: the medial AMG, cortical AMG, and periamygdaloid cortex (Allison, 1954; Nieuwenhuys et al., 2008: Marino et al., 2016; Weiss et al., 2021).

A resting‐state functional magnetic resonance imaging (fMRI) study suggested that the medial AMG is involved in odor‐induced emotional responses, while the cortical AMG is important for learning and memory aspects of these responses, and the periamygdaloid cortex plays a role in olfactory cognition through its connections with the orbitofrontal cortex (OFC) (Noto et al., 2021). It is important to determine how olfactory information is transferred from the olfactory bulb to the AMG, and how it is integrated and organized in higher brain centers.

Olfactory impairment is the first sign of mild cognitive impairment and Alzheimer's disease (Doty et al., 1988; Hawks, 2003; Robert et al., 2016). Impaired olfactory cognition in neurodegenerative disorders may be associated with pathological changes in the AMG and surrounding areas, including the entorhinal cortex (ENT) and hippocampus (HI) (Ubeda‐Bañon et al., 2011). Pathological changes begin in the AMG and HI, and especially in the parahippocampal gyrus (para‐HI) and ENT; these areas play important roles in olfactory detection and recognition (Doty et al., 1988; Hawks, 2003; Mesholam et al., 1998; Ubeda‐Bañon et al., 2011). Olfactory brain volume reductions accompanied by pathological changes, as reflected in olfactory ability, could be useful for predicting disease onset. Therefore, it is important to determine how small structural and functional changes in olfactory ability relate to pathology of the AMG, HI, para‐HI, ENT, and OFC.

Previously, we investigated the relationship between olfactory perceptions and volumes of olfactory‐related regions in healthy elderly subjects, and showed that decreased volume of the HI, para‐HI, and ENT was associated with decreased olfactory recognition ability (Iizuka et al., 2021; Kubota et al., 2020). Functional magnetic resonance imaging (fMRI) showed the relevance of OFC activation to olfaction: olfactory stimuli associated with memory retrieval activated the left OFC in healthy young subjects, and left posterior OFC activation dovetailed with activation of the AMG, para‐HI, and middle frontal cortex (Watanabe et al., 2018). Olfactory discrimination, and the recognition of retrieved memories and emotions, might be associated with activation of the OFC. We aimed to determine how AMG, HI, para‐HI, ENT, and OFC activation relates to olfactory detection (perceiving but not recognizing or identifying an odor) and recognition (identifying an odor and associated emotion) in elderly subjects.

In the present study, we measured brain activation using fMRI during the presentation of olfactory stimuli (classified as “normal odors” not inducing memory retrieval; Watanabe et al., 2018), and investigated the relationship between blood oxygen level‐dependent (BOLD) signals and olfactory detection and recognition ability in healthy elderly subjects. The purpose of the study was to investigate how functional declines in olfactory ability relate to activation of primary olfactory regions, including the AMG, HI, para‐HI, and ENT, as well as OFC subregions (frontal medial OFC, frontal middle OFC, and frontal superior OFC).

## 2 MATERIALS AND METHODS

### 2.1 Participants

The subjects in this study were a subset of those in previous studies (Iizuka et al., 2021; Kubota et al., 2020; Masaoka et al., 2021) with MRI, olfaction, and cognitive test data obtained in 2020. Older adults without cognitive impairment (*N* = 30), as confirmed by two neurologists using the Japanese version of the Montreal Cognitive Assessment (MoCA) (Nasreddine et al., 2005), were tested in this study.

Individuals with a history of head trauma or epileptic seizures, and those with a diagnosis of neurological disease, were excluded. All subjects were living independently; however, 1 had a history of subarachnoid hemorrhage and 5 were unable to undergo fMRI, so that 24 subjects (12 males and 12 females; mean age = 74.4 ± 5.2 years) were finally included in the study.

This study was approved by the Institutional Review Board of Showa University Hospital and the Ethics Committee of Showa University School of Medicine. All participants provided informed consent prior to participation. All experiments were conducted in accordance with the Declaration of Helsinki.

### 2.2 Assessment of olfaction and cognition

The T&T is an olfactory test often used to examine patients with olfactory disorders. Developed in Japan in 1978, the T&T olfactory test was designed to assess olfaction using everyday olfactory stimuli. A detailed description of the test is provided elsewhere (Kubota et al., 2020). In brief, the T&T involves five odors: odor A is “rose” (β‐phenylethyl alcohol), odor B is “burnt” (methylcyclopentenolone), odor C is “sweaty” (isovaleric acid), odor D is “peach” (γ‐undecalactone), and odor E is “feces” (skatole). Each odor is diluted 10 times and divided into eight (−2 to 5) or seven (−2 to 4) concentrations, with 0 being the threshold concentration for normal olfaction. Each trial begins with the lowest concentration, followed by progressively higher ones. During each trial, the subject is asked whether they perceived an odor. The concentration at which an odor is perceived but not identified is considered the “detection level.” As the concentration increases, the subject is more likely to be able to identify the odor. The subject is required to identify and name each odor. The concentration at which an odor is first identified is considered the “recognition level.” Each subject's odor detection threshold is expressed as the average of all odor threshold scores (A + B + C + D + E / 5). The recognition threshold is calculated in the same manner. Higher scores indicate lower olfactory detection and recognition abilities. When using the T&T olfactory test, the degree of olfaction loss is classified as follows: ≤1, “none”; 1.1−2.5, “mild” 2.6−4.0, “moderate”; 4.1−6.0, “severe” or “loss of smell.” The severe and loss of smell classifications are taken to indicate olfactory impairment. All of the olfactory and cognitive data are presented in Supplemental Table S1.

### 2.3 Acquisition of MRI data and olfactory stimuli for fMRI

The imaging protocol used in this study was detailed previously (Masaoka et al., 2021). After a brief clinical examination, MRI scanning (3‐Tesla Magnetom Trio scanner; Siemens, Erlangen, Germany) was conducted at Ebara Hospital, Tokyo, Japan, between 6 pm and 8 pm on Mondays. The scanner had a 32‐channel head coil, and functional images were acquired via slice‐accelerated gradient‐echo echo planar imaging. To increase the temporal resolution, four slices were acquired simultaneously. The fMRI time‐series comprised 330 whole‐brain volumes/session, with each volume comprising 39 axial slices (matrix: 80 × 80; repetition time: 1 s; echo time: 27 ms; field of view: 16−22 cm, thickness: 2.5 mm; flip angle: 90°). Anatomical MRI images were also acquired (3D‐magnetization‐prepared rapid gradient‐echo T1‐weighted sagittal sections).

For the fMRI experiment, odor stimuli were delivered using a previously described, custom‐designed MRI‐compatible system (Masaoka et al., 2014; Watanabe et al., 2018). In brief, each subject wore a nasal mask (ComfortGel Blue Nasal Mask 1070038, medium size; Phillips Respironics, Murrysville, PA, USA), equipped with three inhalation valves with odor cassettes and one exhalation valve. The inhalation valve is a balloon valve that can be opened and closed remotely (i.e., outside of the MRI scanner). When the balloon valve was opened, the odor was delivered to the subject via their force of own inspiration through the odor cassette. The exhaled breath flows through the exhalation valve. The scent is administered for 30 s to prevent olfactory fatigue, and the block design comprises five sets with 30‐s intervals. The experimental setup is described in detail elsewhere (Watanabe et al., 2018).

β‐phenyl ethyl alcohol was used for odor stimuli, as in a previous study (Masaoka et al., 2021), which confirmed that the stimuli did not elicit memory retrieval or arousal (i.e., the stimuli were categorized as “normal odors”) (Masaoka et al., 2012, 2021). Subjects were requested to breath normally and avoid sniffing behaviors during odor presentation. All subjects provided ratings of odor intensity and pleasantness, and the extent of memory retrieval, using visual analogue scales ranging from 0 to 100 mm (anchored by “Unpleasant,” “No odor,” and “No memory retrieval” on the left and “Pleasant,” “Most intense odor,” and “Strongest memory retrieval” on the right, respectively).

### 2.4 MRI data analysis

#### 2.4.1 Extraction of BOLD signals

The images were processed using Statistical Parametric Mapping software (version 12; Wellcome Department of Cognitive Neurology, London, UK) in the MATLAB environment (R2013B; MathWorks Inc., Natick, MA, USA), on a computer running Mac OS X Yosemite. For image preprocessing, motion correction, coregistration of functional and structural images, normalization, physiological noise correction (using the Drifter toolbox of SPM8), and spatial smoothing (6 mm full width at half maximum Gaussian filter) were applied. For each subject, first‐level analysis was performed of the test odors and unscented air.

**FIGURE 1:**
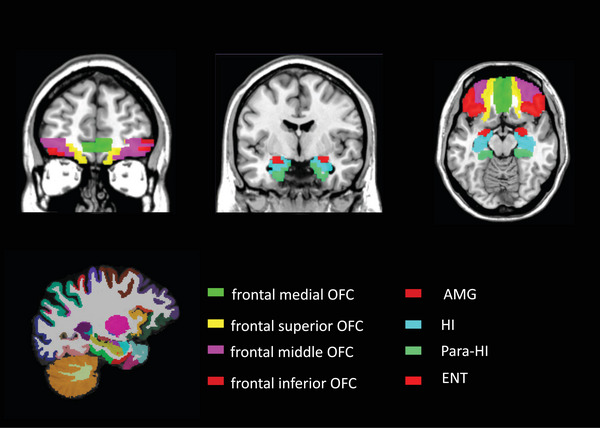
Axial, horizontal, and sagittal sections of olfactory regions of interest in a previous study (Watanabe et al., 2018). AMG, amygdala; HI, hippocampus; para‐HI, parahippocampus; ENT, entorhinal cortex; OFC, orbitofrontal cortex.

Raw mean BOLD signals were extracted from previously described regions of interest (ROIs) (Watanabe et al., 2018), including bilateral regions (AMG, HI, para‐HI, and ENT) and orbitofrontal subregions (frontal inferior OFC, frontal medial OFC, frontal middle OFC, and frontal superior OFC) (Figure 1). We used the Marsbar ROI toolbox (http://marsbar.sourceforge.net/) for SPM8 to create an 8 mm sphere at the Montreal Neurological Institute coordinates (left AMG, *x* = −20, *y* = −2, *z* = −16; right AMG, *x* = 20, *y* = −2, *z* = −16; left HI, *x* = −24, *y* = −14, z = −12; right HI, *x* = 24, *y* = −14, *z* = −12; left para‐HI, *x* = −18, *y* = −32, *z* = −20; right para‐HI, *x* = 18, *y* = −32, *z* = −20; left ENT, *x* = −18, *y* = 6, *z* = 24; right ENT, *x* = 18, *y* = 6, *z* = 24; left frontal inferior OFC, *x* = −32, *y* = 28, *z* = −22; right frontal inferior OFC, *x* = 32, *y* = 28, *z* = −22; left frontal medial OFC, *x* = −14, *y* = 24, *z* = −18; right frontal medial OFC, *x* = 14, *y* = 24, *z* = −18; left frontal middle OFC, *x* = −6, *y* = 60, *z* = −8; right frontal middle OFC, *x* = 6, *y* = 60, *z* = −8; left frontal superior OFC, *x* = −4, *y* = 62, *z* = −2; right frontal superior OFC, *x* = 4, *y* = 62, *z* = −2), with reference to specific primary olfactory cortical and limbic regions described previously (Watanabe et al., 2018 [Supplemental Table S2]).

All BOLD signals, subject information, and olfactory and MoCA scores were entered into SPSS spreadsheets (version 23.0; IBM Corp, Armonk, NY, USA) for analysis. Path analysis was performed with SPSS AMOS (version 23.0; IBM Corp.).

#### 2.4.2 Data analysis

Before the path analysis, partial correlation and multiple regression analyses were performed to refine our hypothesis. All three analyses considered pleasantness, smell intensity, and the extent of memory retrieval. The partial correlation analyzed the relationships of ROIs with olfactory detection, olfactory recognition and MoCA scores after controlling for sex, duration of education, smell intensity and pleasantness, and the extent of memory retrieval. In the multiple regression analysis, odor detection and recognition was the dependent variable, and significant BOLD signals in the partial correlation analysis, pleasantness, smell intensity, and the extent of memory retrieval were the independent variables.

The path analysis further explored the relationships of significant variables in the partial correlation and multiple regression analyses with olfactory detection and recognition. After testing the full model (i.e., that including all paths), we eliminated nonsignificant paths. The goodness of fit of the final model was 0.9 (*p* = .31; Bollen‐Stine bootstrap method).

## 3 RESULTS

The demographic data are shown in Table S1. All of the olfactory and cognitive data are presented in Supplemental Table S1. The decrease in olfactory threshold ranged from none to moderate. The decrease in olfactory recognition ranged from mild to moderate in 20 subjects; the other 4 subjects were in the severe or loss of smell (score of 6) category, and were thus considered to have olfactory impairment. The mean detection and recognition scores of these subjects were lower than those of the age‐matched patients with neurodegenerative disorders in our previous report (Masaoka et al., 2013). All of the subjects in this study had normal MoCA scores, as confirmed by two neurologists. The mean visual analogue scale odor intensity, odor pleasantness, and memory retrieval scores are presented in Table 1. These visual analogue scales were anchored by “No odor,” “Unpleasant odor,” and “No memory retrieval,” respectively (0 mm), and by “Most intense odor,” “Pleasant,” and “Strongest memory retrieval,” respectively (100 mm).

**TABLE 1:** Demographic data of elderly subjects

Number of subjects, sex	24 (female, 12/male, 12)
Age	74.4 ± 5.2 years
Handness	right, 22 / left, 2
MoCA	25.4 ± 2.3
Year of education	13.9 ± 2.6
Olfactory threshold	0.9 ± 0.7
Olfactory recognition level	2.9 ± 1.5
Subjective scales for the odor	
Pleasantness	52.3 ± 19.2
Memory retrieval	31 ± 24.1
Intensity	45.8 ± 27

Mean and standard deviation of the MoCA (Montreal Cognitive Assessment), olfactory threshold and olfactory recognition scores measured with T&T olfactory test (see details in the method), and subjective scales for odor measured with the visual analogue scales ranging from 0 to 100 mm.

**FIGURE 2:**
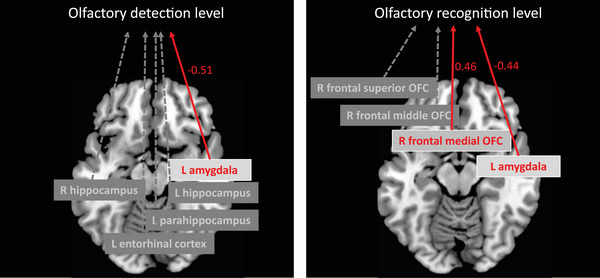
L, left; R, right; AMG, amygdala; HI, hippocampus; para‐HI, parahippocampus; ENT, entorhinal cortex; OFC, orbitofrontal cortex. Results of multiple regression analysis of olfactory detection and recognition. The activity of the left AMG was the most important factor in olfactory detection. The activity of the left AMG and right frontal medial OFC influenced olfactory recognition, albeit in opposite directions.

Table 2 shows the mean BOLD signal for each subject. There were partial correlations between olfactory detection the left AMG (*p* = .02), left HI (*p* = .02), right HI (*p* = .03), left parahippocampus (*p* = .05), and left ENT (*p* = .03) BOLD signals (Table 3). Furthermore, there were partial correlations between olfactory recognition and left AMG (*p* = .02), right medial OFC (*p* = .006), right frontal middle OFC (*p* = .02), and right frontal OFC (*p* = .04) BOLD signals. In multiple regression analysis, the left AMG BOLD signal was negatively associated with olfactory detection (*β* = −0.52, *p* = .01) (Figure 2, left). Olfactory recognition was negatively associated with the left AMG BOLD signal (*β* = −0.42, *p* = .013) and positively associated with the right frontal medial OFC BOLD signal (*β* = 0.57, *p* = .004) (Figure 2, right). Because lower scores for olfactory detection and recognition indicate higher olfactory abilities, the negative associations of the detection and recognition scores with BOLD signals indicate that the subjects with lower olfactory scores had higher activity in brain regions such as the left AMG. The statistical results, including other independent variables, levels of odor pleasantness and intensity, and memory retrieval, are provided in full in Supplemental Table S2.

**TABLE 2:** BOLD signal extracted from olfactory regions of interest

**Brain regions**	**BOLD signal (left/right)**
AMG	0.04 ± 0.46/0.13 ± 0.39
HI	0.08 ± 0.30/0.06 ± 0.19
Para‐HI	0.05 ± 0.22/0.05 ± 0.27
ENT	0.08 ± 0.44/0.11 ± 0.32
frontal inferior OFC	0.09 ± 0.13/0.10 ± 0.13
frontal medial OFC	−0.14 ± 0.37/−0.14 ± 0.37
frontal middle OFC	−0.09 ± 0.23/0.10 ± 0.13
frontal superior OFC	0.09 ± 0.18/0.09 ± 0.20

Mean and standard deviation of BOLD (blood oxygen level‐dependent) signal of olfactory regions, AMG (amygdala), HI (hippocampus), para‐HI (parahippocampus), ENT (entorhinal cortex), OFC (orbitofrontal cortex).

**TABLE 3:** Partial correlation between olfactory detection and recognition levels, MoCA and BOLD signal of ROI

	Olfactory				MoCA	
	detection		recognition			
	*r*	*p*	*r*	*p*	*r*	*p*
L‐amygdala	–0.53	.02	–0.53	.02	0.27	.27
R‐amygdala	–0.39	.1	–0.22	.37	–0.06	.78
L‐hippocampus	–0.52	.02	–0.32	.18	0.03	.86
R‐hippocampus	–0.49	.03	–0.18	.46	0.15	.54
L‐parahippocampas	–0.45	.05	–0.07	.78	0.03	.89
R‐parahippocampas	–0.43	.07	–0.16	.5	–0.04	.81
L‐entorhinal cortex	–0.5	.03	–0.32	.19	0.16	.5
R‐entorhinal cortex	–0.28	.24	–0.14	.56	0.16	.51
L‐frontal inferior orb	–0.18	.47	–0.15	.54	–0.05	.83
R‐frontal inferior orb	0.04	.87	0.14	.56	–0.12	.61
L‐frontal medial orb	–0.12	.65	0.26	.29	0.3	.22
R‐frontal medial orb	0.32	.19	0.62	.006	0.01	.95
L‐frontal middle orb	–0.16	.51	–0.05	.83	–0.37	.13
R‐frontal middle orb	0.32	.19	0.51	.02	–0.34	.16
L‐frontal superior orb	0.09	.71	0.31	.2	–0.05	.83
R‐frontal superior orb	–0.11	.63	0.48	.04	–0.07	.77

Sex, years of education, odor intensity, odor pleasantness, and extent of memory retrieval were entered as covariates. Statistically significant values are indicated as gray squares.

BOLD, blood oxygen level‐dependent; MoCA, Montreal Cognitive Assessment; ROI, regions of interest; L, left; R, right; AMG, amygdala; HI, hippocampus; para‐HI, parahippocampus; ENT, entorhinal cortex; OFC, orbitofrontal cortex.

**FIGURE 3:**
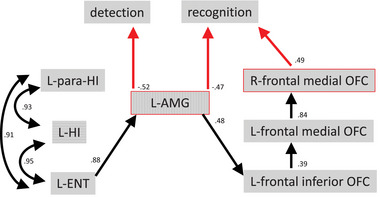
Path diagram showing significant direct paths between the left amygdala and olfactory detection and recognition abilities. L, left; R, right; AMG, amygdala; HI, hippocampus; para‐HI, parahippocampus; ENT, entorhinal cortex; OFC, orbitofrontal cortex. The right frontal medial OFC also had significant paths. Left AMG activation may be crucial for both olfactory detection and recognition. In contrast, less activation of the OFC was associated with better olfactory recognition.

On the basis of the above results, we performed a path analysis to investigate how olfactory detection and recognition interact with each other through the activation of olfactory limbic regions and frontal areas. Figure 3 shows significant path and coefficient values between brain regions and olfactory detection and recognition. All direct and indirect path coefficient values are provided in Supplemental Tables S3 and S4.

Left AMG activity, which affected olfactory detection abilities, was influenced by left ENT activity, which was in turn strongly correlated with left para‐HI and left HI activity. The left ENT had an indirect effect on olfactory detection (−0.45) and recognition (−0.35), as well as left frontal inferior OFC activity (0.4).

Among the sites implicated in olfactory detection, the left AMG also impacted olfactory recognition. The left AMG had a positive effect on left frontal inferior OFC activity. The left frontal inferior OFC had a significant direct path to the left frontal medial OFC, and the left frontal medial OFC had a strong impact on frontal medial OFC activity. Finally, the frontal medial OFC was positively associated with olfactory recognition.

Interestingly, strong activation of the left AMG was associated with good olfactory detection and recognition, which involved an indirect path from the left ENT (Supplemental Table S4). Weak activation of all OFC subregions was also associated with good olfactory recognition. The effects of OFC activation and AMG/ENT activation on olfactory recognition were in the opposite direction.

## 4 DISCUSSION

In this study, the mean scores for olfactory detection and recognition were in the normal range; however, interindividual variability was observed in the level of brain activity in the AMG and OFC regions, and the associations thereof with olfactory detection and recognition scores.

Among brain areas, activation of the left AMG had the greatest impact on olfactory detection and recognition. In contrast, less activation of the right frontal medial OFC was associated with good olfactory recognition. These opposing effects shed light on the associations of activity in limbic and prefrontal regions with olfactory awareness and identification in elderly individuals.

### 4.1 Role of the AMG in olfactory detection and recognition

We confirmed that activation of the AMG plays an important role in olfactory detection and recognition. Previous studies showed that olfactory decline in elderly subjects was associated with reduced volume of the left HI, left para‐HI, and ENT (Iizuka et al., 2021; Kubota et al., 2020). Furthermore, a clinical study showed that pathological changes in the ENT and para‐HI impaired the olfactory ability of Alzheimer's and mild cognitive impairment patients (Koychev et al., 2017). On the basis of these studies, we suspected that activation of the ENT and para‐HI might be important for olfactory ability; in fact, the AMG had the greatest impact on olfactory detection and recognition, with the ENT, para‐HI, and HI acting as a support system for AMG activation. It has been reported that, unlike the ENT and para‐HI, the volume of the AMG remains unchanged in the elderly (Iizuka et al., 2021; Kubota et al., 2020). AMG activation may serve as a hub integrating information from the ENT, para‐HI and HI. The ENT acts as a “gateway” to the HI, allowing for memory retrieval (Amaral et al., 1987), while the para‐HI is involved in memory processing (Naya, 2016). Overall, the data indicate that information allowing for odor detection and recognition is organized within the AMG.

### 4.2 Role of the frontal medial OFC in olfactory recognition

Left AMG activation was associated with odor recognition in this study, and showed a negative association with olfactory recognition. Olfactory information is transmitted through direct projections from the olfactory bulb to the primary olfactory cortex, including the anterior olfactory cortex, ventral tenia tecta, anterior hippocampal continuation, indusium griseum, olfactory tubercle, piriform cortex, anterior cortical nucleus of the AMG, periamygdaloid cortex, and rostral ENT (Yeshurun & Sobel, 2010).

Olfactory information transmitted through primary olfactory regions converges in the OFC, allowing for cognitive discrimination (Rolls, 2004). These olfactory projections are similar to those involved in emotion regulation. Previous functional connectivity analyses showed that OFC and rostral anterior cingulate cortex activity were positively correlated with AMG activity during emotion regulation (Banks et al., 2007; Erk et al., 2006; New et al., 2007). Our results showed that the BOLD signal of the AMG positively correlated with frontal OFC activity during olfactory recognition. Olfactory inputs from the AMG directly project to the frontal inferior OFC (Mesulam & Mufson, 1982), which collaborates with other structures in the prefrontal cortex, including the frontal medial OFC (Barbas & Pandya, 1989). As expected, there was positive correlation in activity among these areas in this study, although increased OFC activity was not associated with good olfactory performance.

The frontal cortex has a top‐down inhibitory effect on the AMG (Pears et al., 2003; Quirk & Beer, 2006), where prefrontal projections inhibit neurons in the latter structure (McDonald et al., 1996). During emotion regulation, AMG activity is controlled by the prefrontal cortex (Davidson et al., 2000). In particular, the frontal medial OFC plays an important role in attentional and cognitive control (Brefczynski‐Lewis et al., 2007). Against this background, interactions of the frontal medial OFC with the AMG may play a role in olfactory recognition; less inhibition of the AMG by the frontal medial OFC may improve cognitive processing in relation to olfaction. We observed that weak activation of the AMG was associated with low olfactory detection ability. Therefore, strong activation of the AMG, in conjunction with activation of the ENT, para‐HI and HI, could be important for the identification and/or recognition of odors, and mild activation of frontal regions may aid interpretation of olfactory information. In other words, AMG‐frontal medial OFC‐mediated regulation of the olfactory system may represent a bottom‐up process of integration of olfactory stimuli. Top‐down integration within the olfactory system is a potential target for future research, which could assess whether prior olfactory information conveyed by words affects AMG activation.

### 4.3 Laterality

Laterality in the olfactory system has been discussed in a number of studies. An fMRI study reported that odor‐induced emotions activated the right OFC (Gottfried et al., 2002), while in another study the left OFC was activated during the presentation of an unpleasant odor (Rolls et al., 2003).

The left posterior OFC has a dominant role in odor memory recognition (Masaoka et al., 2021), while the left OFC is dominant in terms of odor‐related emotion processing (Royet et al., 2003). In this study, left hemisphere structures including the AMG, ENT, para‐HI, HI were involved in olfactory detection. The left primary olfactory regions were crucial for odor recognition, in line with studies on structural volume changes demonstrating that a decline of olfactory ability was associated with left HI, para‐HI, and ENT volume reductions (Kubota et al., 2020; Iizukua et, al., 2021). As well as structural volume decreases in the left HI, para‐HI, and ENT, reduced functional activity in these areas affects olfactory detection and recognition. Interestingly, we observed that the left AMG has a direct anatomical connection to the left front inferior OFC, and activation shifted to the right in our study. The inferior frontal gyrus includes the posterior part of the OFC, which plays an important role in integrating odor information (Du et al., 2020) and may mediate communication between the temporal regions and OFC (Sinding et al., 2021). Odor information processing within the OFC is poorly understood, but the relationship between the left AMG and inferior frontal OFC could be important; inhibitory actions within the bilateral OFC may also play a role.

### 4.4 Limitations and future research

Several limitations of this study should be noted. First, we included a small number of elderly subjects; larger studies including younger subjects are needed. Second, we included only one odor previously used as a control odor, that is, an odor not associated with emotional arousal or memory retrieval. Although we analyzed odor intensity and pleasantness, as well as memory retrieval, other factors and subjective feelings might have influenced the results. Previously, it was shown that AMG activation was related to odor intensity (Anderson et al., 2003; Winston et al., 2005). Interindividual variability in comfortable odor intensity may merit investigation in future research. Finally, we did not use odors spanning the entire pleasantness scale (i.e., from lowest to highest pleasantness), which should be a target for future studies. Meta‐analysis study in olfaction showed that pleasant odor activated both right and left AMG (Torske et al., 2022). Activation of the AMG by pleasant odors might depend on complex interactions among odor intensity, arousal (Sorokowska et al., 2016), and odor memory; future research aiming to understand how interactions between the AMG and frontal regions influence odor processing should take this into account.

### 4.5 Summary

This study showed that the AMG plays a central role in olfactory detection and recognition. Activation of the AMG and OFC had opposite effects on olfactory recognition, and their interaction (through both excitatory and inhibitor mechanisms) may be important for olfactory perception. The AMG regulates attention to emotional stimuli, and can enhance sensory processing by reducing the activity of neurons in the sensory cortex through cholinergic neurons excited by the AMG (Ohira, 2004; Whalen et al., 1998). This suggests that excitation of the AMG may reduce the activity of the frontal medial OFC, as an alternative mechanism to inhibitory control of the AMG by frontal regions.

In patients with mild cognitive impairment, pathological changes first occur in the ENT and para‐HI. Ubeda‐Bañon et al. (2011) suggested that the ENT, HI, and AMG form a key interconnecting network for the olfactory system. Functional decline of these structures may be crucial for olfactory recognition; however, the AMG may compensate for this, and interactions between the AMG and frontal region are also important for olfactory identification.

## AUTHOR CONTRIBUTIONS

YM and KS designed the study. KS, YM, and MH performed the statistical analysis. KS, YM, AY, MY, SK, and MI conducted all experiments. YM, KS, HK, and MI were responsible for drafting the manuscript. All authors approved the publication of the study, and agreed to be accountable for all aspects of the work and to ensure that questions related to the accuracy or integrity of any aspect thereof are appropriately investigated and resolved.

## CONFLICT OF INTEREST STATEMENT

The authors declare that the study was conducted in the absence of any commercial or financial relationships that could be construed as a potential conflict of interest.

### PEER REVIEW

The peer review history for this article is available at https://publons.com/publon/10.1002/brb3.2956.

## Supporting information

Supp Information

## Data Availability

Data sets generated for this study are available on request from the corresponding author.
