# Supplementary material for: Role of the amygdala‐medial orbitofrontal relationship in odor recognition in the elderly
Source: Brain Behav. 2023 Mar 10;13(4):e2956. doi: 10.1002/brb3.2956 (PMC10097066; doi:10.1002/brb3.2956)
Supplement: Supplementary file 1 — Supp Information [file BRB3-13-e2956-s001.docx]

**Supplemental Materials**

**Role of the amygdala-medial orbitofrontal relationship in odor recognition in the elderly**

Running title: Amygdala-orbitofrontal cortex and olfaction

Kei Sakikawa^1,2^, Yuri Masaoka^1†^*, Motoyasu Honma^1^, Akira Yoshikawa^1,3^, Masaki Yoshida^4^, Sawa Kamimura^1,2^, Masahiro Ida^5^, Hitome Kobayashi^2^, Masahiko Izumizaki^1^

1. Department of Physiology, Showa University School of Medicine, Tokyo, Japan
2. Department of Otorhinolaryngology Head and Neck Surgery, Showa University School of Medicine, Tokyo, Japan
3. Division of Health Science Education, Showa University School of Nursing and Rehabilitation Sciences, Yokohama, Japan
4. Department of Ophthalmology, Jikei Medical University, Tokyo, Japan
5. Department of Radiology, National Hospital Organization Mito Medical Center, Ibaraki, Japan

^†^The authors contributed equally to this work

***Corresponding Author:**

Yuri Masaoka (faustus@med.showa-u.ac.jp)

Department of Physiology, Showa University School of Medicine

1-5-8 Hatanodai, Shinagawa-ku, Tokyo 142-8555, Japan

Tel: +81-3-3784-8113

Fax: +81-3-3784-0200

Supplemental Table 1 Olfactory and cognitive data of all subjects

| Subject No. | MoCA | Olfactory threshold | Olfactory recognition |
| --- | --- | --- | --- |
| 1 | 23 | -0.4 | 2.6 |
| 2 | 20 | 0 | 3.4 |
| 3 | 24 | 0.2 | 0.8 |
| 4 | 26 | 0.2 | 1.6 |
| 5 | 27 | 0 | 3 |
| 6 | 25 | 0.8 | 2.8 |
| 7 | 26 | 1 | 2 |
| 8 | 28 | 0.8 | 1.8 |
| 9 | 27 | 1.2 | **6** |
| 10 | 28 | 0.8 | 2.4 |
| 11 | 27 | 1.2 | 3.6 |
| 12 | 24 | 1 | 2 |
| 13 | 28 | 1.2 | 1.8 |
| 14 | 22 | 1.8 | 3 |
| 15 | 27 | 2 | 3.6 |
| 16 | 27 | 1 | **6** |
| 17 | 23 | 1.4 | 2 |
| 18 | 24 | 0.4 | 1.8 |
| 19 | 26 | 0.4 | 2.4 |
| 20 | 24 | 1 | 2.4 |
| 21 | 26 | 1.4 | **6** |
| 22 | 29 | 0.4 | 1.8 |
| 23 | 26 | 1.6 | 2.2 |
| 24 | 22 | 3 | **6** |
| Average | 25.4 | 0.9 | 2.9 |
| Standard Deviation | 2.3 | 0.7 | 1.5 |

The T&T involves five odors (for details, see main text). Each odor is diluted 10 times and divided into eight (-2 to 5) or seven (-2 to 4) concentrations. Each trial begins with the lowest concentration, which is then progressively increased until the highest concentration is reached. During each trial, the subject is asked whether they perceived an odor. The concentration at which an odor is perceived but not identified is considered the “detection level.” As the concentration increases, the subject is more likely to be able to identify the odor. The subject is required to identify and name each odor. The concentration at which an odor is first identified is considered the “recognition level.” Each subject’s odor detection threshold is expressed as the average of all odor threshold scores (A + B + C + D + E / 5). The recognition threshold is calculated in the same manner. Higher scores indicate lower olfactory detection and recognition abilities. An odor recognition score of 6 is taken to indicate severe olfactory impairment.

Supplemental Table 2 The statistical results of multiple regression, including other independent variables, levels of odor pleasantness and intensity, and memory retrieval.

Olfactory detection

The left AMG BOLD signal was negatively associated with olfactory detection (β = -0.52, p = 0.01). The other areas and subjective scales were not associated with olfactory detection.

Olfactory recognition was negatively associated with the left AMG BOLD signal (β = -0.42, p = 0.013) and positively associated with the right frontal medial OFC BOLD signal (β = 0.57, p = 0.004). The other areas and subjective scales were not associated with olfactory recognition.

Supplemental Table 3 All standardized direct path coefficient values.

| Brain regions | | Estimate | P |
| --- | --- | --- | --- |
|  |  |  |  |
| L ENT | L AMG | 0.88 | P<0.001 |
| L AMG | olfactory detection | -0.52 | 0.004 |
| L AMG | olfactory recognition | -0.47 | 0.004 |
| L AMG | L frontal inferior OFC | 0.48 | 0.012 |
| L frontal inferior OFC | L frontal medial OFC | 0.39 | 0.04 |
| L frontal medial OFC | R frontal medial OFC | 0.84 | P<0.001 |
| R frontal medial OFC | olfactory recognition | 0.49 | 0.003 |
|  |  |  |  |
| Covariance correlation |  |  |  |
| Brain regions | | Estimate | P |
| L para-HI | L ENT | 0.91 | 0.001 |
| L para-HI | L HI | 0.92 | 0.001 |
| L HI | L ENT | 0.95 | P<0.001 |

Path analysis was to investigate how olfactory detection and recognition interact with each other through the activation of olfactory limbic regions and frontal areas. Direct path coefficient values between two brain regions were indicated.

L, left; R, right; ENT, entorhinal cortex; AMG, amygdala; OFC, orbitofrontal cortex; para-HI, parahippocampus; HI, hippocampus

Supplemental Table 4 All standardized indirect path coefficient values.

| Brain regions | | Estimate |
| --- | --- | --- |
| L ENT | olfactory detection | -0.45 |
| L ENT | olfactory recognition | -0.35 |
| L ENT | L frontal inferior OFC | 0.4 |
| L ENT | L frontal medial OFC | 0.15 |
| L ENT | R frontal medial OFC | 0.13 |
|  |  |  |
| L AMG | L frontal medial OFC | 0.17 |
| L AMG | R frontal medial OFC | 0.14 |
| L AMG | olfactory recognition | 0.15 |
|  |  |  |
| L frontal inferior OFC | R frontal medial OFC | 0.32 |
| L frontal inferior OFC | olfactory recognition | 0.15 |
| L frontal medial OFC | olfactory recognition | 0.4 |

Indirect path coefficient values were indicated. The indirect effect is a relationship that flows from an independent variable to a mediator and then to a dependent variable. For example in this table, the relationship flow from the L ENT to the L AMG and then to the detection (please see an arrow indicated in Figure 3). However, all indirect path were not significant. Direct path between variables were impacted in this model.

L, left; R, right; ENT, entorhinal cortex; AMG, amygdala; OFC, orbitofrontal cortex; para-HI, parahippocampus; HI, hippocampus
